# The Tie2 signaling pathway in retinal vascular diseases: a novel therapeutic target in the eye

**DOI:** 10.1186/s40942-020-00250-z

**Published:** 2020-10-13

**Authors:** Quan Dong Nguyen, Jeffrey S. Heier, Diana V. Do, Adam C. Mirando, Niranjan B. Pandey, Huan Sheng, Theresa Heah

**Affiliations:** 1 Spencer Center for Vision Research, Byers Eye Institute, Stanford University, 2370 Watson Court, Suite 200, Palo Alto, CA 94303 USA; 2 Ophthalmic Consultants of Boston, Boston, MA USA; 3 AsclepiX Therapeutics, Baltimore, MD USA

**Keywords:** Tie2, Integrin, Vascular endothelial grwoth factor, Vascular permeability, AXT107

## Abstract

**Background:**

Retinal vascular diseases such as neovascular age-related macular degeneration, diabetic retinopathy and/or diabetic macular edema, and retinal vein occlusion with macular edema—share several key pathophysiologic aspects including neovascularization, vascular permeability, and inflammation. The role of vascular endothelial growth factor (VEGF) in these processes, and the therapeutic benefits of VEGF inhibition, have been well characterized. Anti-VEGF therapy is highly effective for many patients but is not uniformly effective in all patients and imposes a significant treatment burden. More recently, the role of the Tie2 signaling pathway in the pathophysiology of retinal vascular diseases has been investigated, and the Tie2 pathway represents a novel therapeutic target for these conditions.

**Areas covered:**

The index review describes the Tie2 pathway and its complementary role to the VEGF pathway in the angiogenesis cascade and will summarize studies of molecules in development to therapeutically modulate the Tie2 pathway in retinal vascular diseases.

**Conclusions:**

Activation of the Tie2 pathway leads to downstream signaling that promotes vascular health and stability and decreases vascular permeability and inflammation. AXT107 is a collagen IV–derived synthetic peptide with a dual mechanism of action that involves suppression of VEGF signaling and activation of the Tie2 pathway; these actions are accomplished by AXT107 binding to and disrupting different integrin, leading to blockade of the VEGF receptor and rearrangement of cellular Tie2 rendering it susceptible to Ang2 agonism. Other Tie2 agonist compounds are also in development, including faricimab and razuprotafib. Tie2 activation only modestly impacts angiogenesis on its own but significantly potentiates VEGF suppression. Co-regulation of the VEGF and Tie2 signaling pathways has the potential to improve functional and structural outcomes in eyes with retinal vascular diseases.

## Background

### Retinal vascular disease in the anti-VEGF Era

The tissues of the eye in general, and the retina more specifically, are highly metabolically active and thus richly vascularized. Consequently, the retina is subject to both primary and secondary vascular diseases. Common retinal vascular disorders include neovascular age-related macular degeneration (nAMD), diabetic retinopathy (DR) and/or diabetic macular edema (DME), and retinal vein occlusion (RVO). While these various entities have distinct, complex, and multifactorial pathogeneses and risk factors, they share several key aspects of pathophysiology, specifically uncontrolled neovascularization, increased vascular permeability, and unregulated inflammation.

The development of therapies that inhibit vascular endothelial growth factor (VEGF) has revolutionized the management of retinal vascular diseases. In the presence of hypoxia, a state common to all ischemic retinal vascular diseases, VEGF synthesis is induced [1]. VEGF is a growth factor that plays a key role in angiogenesis and vascular permeability through its interaction with the VEGF receptor, a receptor tyrosine kinase [2]. VEGF levels are elevated in the aqueous and/or vitreous humor of eyes with nAMD, DR, DME, and RVO [3–5]. Multiple anti-VEGF therapies have been approved for the treatment of retinal vascular diseases—including ranibizumab, aflibercept, and brolucizumab—and another, bevacizumab, is often used off-label as well (Table 1) [6–9].

**Table 1:** Anti-VEGF drugs and their approved indications

Drug	Approved indications
Ranibizumab [6]	nAMD, DR, DME, RVO, myopic CNV
Aflibercept [7]	nAMD, DR with DME, DME, RVO
Brolucizumab [8]	nAMD
Bevacizumab [9]	None (but often used for all of the above)

## Areas Covered

The availability of anti-VEGF therapies has initiated a paradigm shift in the management of retinal vascular diseases. Yet, there remain unmet needs for the treatment of these conditions. The current pharmacologic agents are indicated for repeated intravitreal (IVT) injections every 1–3 months [6–8], although less often retreatment has shown to be effective [10–15]. This significant treatment burden is unsustainable, and many patients receive fewer than the recommended number of injections with commensurately suboptimal visual outcomes [16–21]. More importantly, a proportion of eyes fail to attain optimal visual outcomes even when receiving anti-VEGF therapy at recommended intervals [22, 23].

Such suboptimal outcome is likely because VEGF is only one component of the complex pathophysiology of retinal vascular diseases. Recent research has clarified the critical role of the Tie2 and other pathways—including those related to oxidative stress [24], the complement pathway [25], and others [26]—in the development of retinal vascular diseases including nAMD, DR/DME, and RVO [27–29]. There has been significant work to evaluate the Tie2 pathway. The Tie2 signaling pathway, working in concert with the VEGF signaling pathway, represents a novel therapeutic target for these conditions. Several candidate drugs are in various stages of development, including AXT107, faricimab, and razuprotafib (Table 2). Among these, AXT107 is a collagen IV–derived peptide which acts as an inhibitor of vascular endothelial growth factor receptor 2 (VEGFR2) *and* an activator of the Tie2 pathway with potential therapeutic activity in nAMD, DME, RVO, and other retinal vascular diseases.

**Table 2:** Investigational drugs targeting Tie2 pathway

	AXT107	Faricimab	Razuprotafib	Nesvacumab
Sponsors	AsclepiX	Roche	Aerpio	Regeneron
Structure	Peptide	Bispecific antibody	Small molecule	Monoclonal antibody
Biological effect	Inhibit VEGFR-A/-C Activate Tie2	Inhibit VEGFR-A and Ang-2	Activate Tie2	Inhibit Ang-2
Molecular weight (kDa)	2.357	150	0.587	144.9
Route and Dose frequency	IVT every 6-12 months	IVT every 3-4 months	Subcutenous twice a day	IVT every 4 weeks
Development status	IND filing late 2020 for DME, nAMD and RVO	Phase 3 underway for nAMD and DME	Phase 2 for DME completed	Development terminated

The index review will describe the Tie2 pathway and its role in the pathophysiology of retinal vascular diseases. Current drugs in development will also be reviewed, and the potential roles of these drugs that target the Tie2 pathway in the comprehensive management of retinal vascular diseases will be discussed.

### Overview of the Tie2 pathway

Angiopoietin-1 (Ang1) and angiopoietin-2 (Ang2) are peptide ligands for the tyrosine kinase with Ig (immunoglobulin) and EGF (epidermal growth factor) homology domains 2 (Tie2) receptor [30]. Ang1 is a Tie2 receptor agonist while Ang2 is a contextual antagonist, blocking Tie2 signaling in most contexts but capable of enhancing Tie2 signaling under specific conditions (discussed below). The Tie2 signaling pathway is specific to vascular endothelial cells [30–33] as is the VEGF pathway. However, while the VEGF pathway promotes the *initiation* of angiogenesis, the Tie2 signaling pathway is more active in *maintenance* of vascular health, promoting endothelial cell (EC) survival, maturation, and stability [34].

### Key components of the Tie2 pathway

There are 5 key components of the Tie2 pathway relevant to ocular angiogenesis: the Tie1 and Tie2 receptors, angiopoietin-1 and -2, and the vascular endothelial tyrosine phosphatase (VE-PTP) receptor.

Tie1 and Tie2 are related transmembrane receptor tyrosine kinases that share 76% homology in their intracellular domains and 33% homology in their extracellular domains [35]. These receptors are expressed primarily on vascular endothelial cells (ECs), and can also be found on some hematopoietic cells. The extracellular portion of both receptors consist of Ig-like and fibronectin type III domains, and the intracellular portions are split tyrosine kinase domains. Tie2 binds both Ang1 and Ang2 at its extracellular Ig-like domains [35]; Tie1 does not bind the angiopoietins and its functional role in Ang1/Ang2 signaling is unclear [36].

Ang1 and Ang2 are Tie2 receptor ligands; both bind to Tie2 at the same site with similar affinity [37]. Ang1 is a Tie2 receptor agonist expressed by smooth muscle cells, pericytes and fibroblasts [33, 38]. Ang2 is a Tie2 receptor antagonist in resting ECs [39, 40], although it may also serve as a partial agonist when ECs are in activated or stressed states [41] such as in the setting of inflammation [42]. Ang2 is expressed primarily in ECs [38]. While both Ang1 and Ang2 form clusters, the Ang1 clusters contain greater number of molecules. The greater number of Ang1 molecules results in more extensive aggregation of Tie2 than what Ang2 can achieve, which is important for the strong activation of Tie2 [43–45].

VE-PTP is a transmembrane receptor tyrosine phosphatase that dephosphorylates Tie2 and downregulates Tie2 signaling [46, 47]. Inhibition of VE-PTP promotes Tie2 activation by maintaining Tie2 in the active, phosphorylated state [48].

### Functions of the Tie2 pathway

**Fig. 1:**
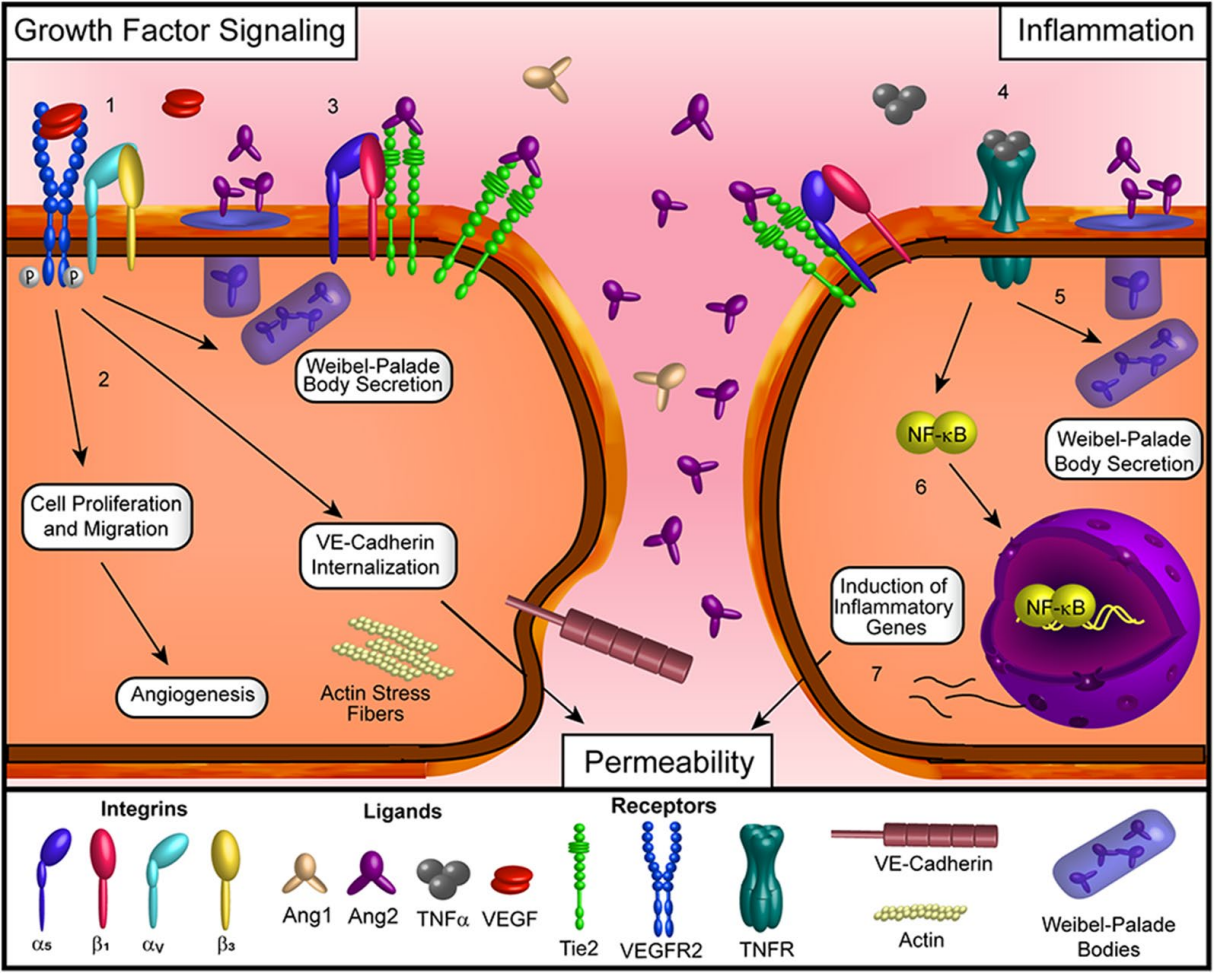
VEGF and TNF signaling in retinal vascular disease. Increased expression of angiogenic and inflammatory factors (such as VEGF and TNFα) are associated with retinal vascular diseases. (1) The binding of VEGF to VEGFR2 in complex with α_v_β_3_ integrin stimulates the autophosphorylation of VEGFR2 and the activation of downstream cellular processes. (2) These processes include the induction of migration and proliferation important for angiogenesis and the reorganization of diffuse actin into stress fibers and the internalization of cell–cell adhesion proteins that result in increased permeability. (3) VEGFR2 signaling also stimulates the release of Ang2 from Weibel-Palade bodies, which then competes with Ang1 to bind to Tie2 and reduce the associated signaling pathways related to vascular stability and anti-permeability. (4) With respect to inflammatory signaling, the binding of TNFα to the TNFR also stimulates the release of Ang2 from Weibel-Palade bodies (5) and reduces the activation of Tie2. This not only reduces the vessel stabilizing pathways mentioned above but also blocks the anti-inflammatory activity of the Tie2 receptor as well. (6) The activation of the TNFR pathway by TNFα also stimulates the relocation of NF-κB to the nucleus where it functions as a transcription factor to induce the expression of numerous inflammation-associated genes. (7) Among other functions, the initiation of inflammation stimulates increased permeability within blood vessels

Ang1 binding to the Tie2 receptor activates Tie2 signaling (Fig. 1). The process begins with clustering and phosphorylation of the Tie2 receptor, which activates several downstream signaling pathways that support EC health. Akt (or protein kinase B) activation promotes EC survival and preservation [49, 50], suppresses Ang2 production via inactivation of transcription factor FOXO1 [51] and promotes vascular stability in EC [52, 53]. The phosphatidylinositol 3-kinase (PI3K) pathway is also activated, promoting EC migration [54]. NF-κB mediated inflammation is suppressed by Tie2 activation [55]. EC permeability is also decreased, and overall EC health is supported and maintained through other incompletely characterized signaling pathways [54]. Ang2 binding disrupts the Tie2 clusters formed by Ang1 and blocks its pro-vascular stability effects. Consequences include abnormal vascular structure formation [56], increased vascular permeability [56], and increased inflammation [57].

Animal studies have demonstrated these effects well. Tie2 deficient mice are non-viable in the second week of gestation; a rudimentary vascular system appears but fails to remodel or mature, with few branches, pericytes, or smooth muscle cells [58, 59]. Ang1-deficient mice share this phenotype with Tie2-deficient mice [60], while overexpression of Ang1 in skin cells produces hypervascularity (vessels are more numerous, larger, and more highly branched) [61] with decreased vascular permeability [62]. Ang2-deficient mice survive but have altered vascularization (abnormal retinal vascularization, failure of regression of hyaloid vessels of the crystalline lens) [63], while overexpression of Ang2 disrupts vascular formation leading to mid-gestational death similar to Ang1 deficiency [32].

### Role of the Tie2 pathway in retinal vascular diseases

The various retinal vascular diseases are characterized by posterior segment neovascularization and/or vasculopathy followed by fluid extravasation into tissues from these permeable new or impaired vessels and are frequently associated with inflammation. Several lines of research demonstrate a pathophysiological role for the Ang-Tie pathway in these conditions.

#### Neovascularization

Choroidal neovascularization is a hallmark of nAMD. Ang2 is a susceptibility gene for the development of nAMD [64], and Ang2 levels are elevated in aqueous humor of eyes with nAMD and correlate with severity of disease [65]. Both Ang1 and Ang2, as well as VEGF, are expressed on excised subfoveal membranes from eyes with nAMD [66]; so is Tie2, and the highest levels of Ang2 and VEGF are from the most highly vascularized regions of these CNV membranes [67]. Preretinal and retinal neovascularization are hallmarks of PDR and RVO, and vitreous levels of Ang2 are elevated in eyes with PDR [68] and RVO [69].

These findings illustrate that ocular neovascularization is driven in part by Tie2 receptor antagonism by Ang2. Conversely, Ang1 suppresses choroidal neovascularization (CNV) and vascular leakage in a laser-induced mouse model [70], demonstrating the potential therapeutic effects of targeting the Tie2 pathway to block the neovascularization process.

#### Vascular permeability

Leakage of fluid into and under the retina is a hallmark of many retinal vascular diseases. Vascular permeability is increased in these eyes through the combined activity of the VEGF and Tie2 pathways. Ang2 potentiates the action of VEGF; VEGF-induced vascular permeability triples in the presence of Ang2 [71]. Ang2 is upregulated in settings of hypoxia [72], hyperglycemia [73, 74] and oxidative stress [75], which are common in eyes of diabetic patients, and Ang2 levels are elevated in the vitreous of diabetic eyes [76, 77], particularly those with retinopathy [78]. Ang2 is increased in sepsis [79, 80]—in which increased vascular permeability is a crucial component—and Ang2 levels correlate with the severity of sepsis [80]. In contrast, Ang1 functions as an anti-permeability growth factor and promotes EC remodeling to minimize permeability [81]. Likewise, Tie2 signaling decreases vascular permeability by tightening intra-EC junctions; this effect is mediated by associations of Tie2 receptors on adjacent cells and reorganization of key adhesion molecule vascular endothelial-cadherin (VE-cadherin) complexes [82]. As examples of its critical role in regulating vascular permeability in health and disease, Tie2 activation reduces pulmonary vascular leakage during cardiopulmonary bypass in rats [83], reduces cerebrovascular permeability and stroke size in mouse models [84], and reduced renal vascular edema and transcapillary albumin flux is a mouse model of acute kidney injury [85].

#### Inflammation

Increased expression of pro-inflammatory cytokines in the retina has been observed in retinal vascular diseases [86–88]. TNFα is a primary cytokine involved in inflammatory disease of the eye. Ang2 can potentiate the activities of TNFα. Under inflammatory conditions, Ang2 is rapidly secreted from EC and antagonizes the vessel-stabilizing activities of the Tie2 receptor by blocking its interaction with Ang1 [89].

The pathophysiology of macular edema, and its adverse effect on visual acuity, is driven by both the VEGF and Tie2 pathways, as well as synergistic interplay between the two. Ang1 blocks VEGF-induced vascular permeability without inhibiting VEGF-induced angiogenesis signalling [90]. The mechanism for the crosstalk responsible for regulating vascular permeability involves the sequestration of cell adhesion molecule VE-cadherin [91], which under normal conditions is located at cell junctions and maintains them but in the presence of VEGF is internalized in intracellular vesicles resulting in cell junction breakdown and increased permeability. Ang1 blocks VEGF-induced redistribution of VE-cadherin, thus preserving EC junctions and preventing increased permeability, through a series of complex interactions with Src-family kinases, Rho-family GTPases, and the formin mDia [90]. This crosstalk between the two pathways can be exploited therapeutically, as Tie2 activation suppresses VEGF-induced vascular leakage and reduces inflammation in mouse models [92, 93].

### Targeting the Tie2 pathway for retinal vascular diseases

#### AXT107

Angiogenesis is a complex multi-step process that requires remodeling of the ECM to make way for vascular growth and to support new vessels. The ECM plays a key regulatory role in all aspects of angiogenesis, including structural support for cells as well as molecular signaling for vascular sprouting, lumen formation, and vessel maturation and stability [94]. ECM degradation liberates numerous small peptides—including angiostatin, endostatin, and several small peptides derived from type IV collagen—that have anti-angiogenic properties and serve as natural regulators of the angiogenesis process [95, 96].

Numerous peptides based on ECM proteolysis products have been synthesized and screened in vitro for anti-angiogenic activity using a computational-based peptidomics approach [97]. Of these, several peptides derived from type IV collagen such as cyrostatin, the wispostatin family, and the chemokinostatin family have potent anti-angiogenic activity [97].

AXT107 (Table 2) is a 20-mer synthetic peptide derived from the non-collagenous domain of collagen IV that may be a triple threat to the pathophysiology of retinal vascular diseases: suppressing ocular neovascularization, inhibiting vascular leakage, and reducing inflammation [79, 92]. AXT107 targets two pathways in a single molecule: it promotes Tie2 pathway signaling [82] while blocking VEGF pathway signaling [92]. Both of these actions are mediated by AXT107’s interaction with and disruption of the proteins integrin α_v_β_3_ [82, 92] and integrin α_5_β_1_ [82, 92, 98–100].

Integrins are essential for the attachment of cells to the ECM. Integrin α_5_β_1_ interacts with Tie2 receptors and sequesters them at EC-ECM interface [82]. In this resting state, Ang2 is a Tie2 antagonist. In the presence of AXT107, however, integrin α_5_β_1_ is disrupted and Tie2 receptor molecules can then reorganize within cells, clustering at EC–EC junctions. These clusters are presumed to be similar to the Tie2 superclusters that form naturally in response to Ang1 exposure but are inactive in absence of a bound ligand. However, by precluding the requirement of the ligand to form these structures, either Ang1 or Ang2 are able to bind and activate Tie2 signaling. The reorganization and junctional clustering of Tie2 induced by AXT107 allows Ang2, which normally functions as an antagonist of Tie2 and is an inducer of vascular leakage, to function as a strong agonist of Tie2 and a promoter of vascular integrity. Tie2 phosphorylation then activates the myriad downstream signaling pathways that promote vascular integrity and health [82, 92].

**Fig. 2:**
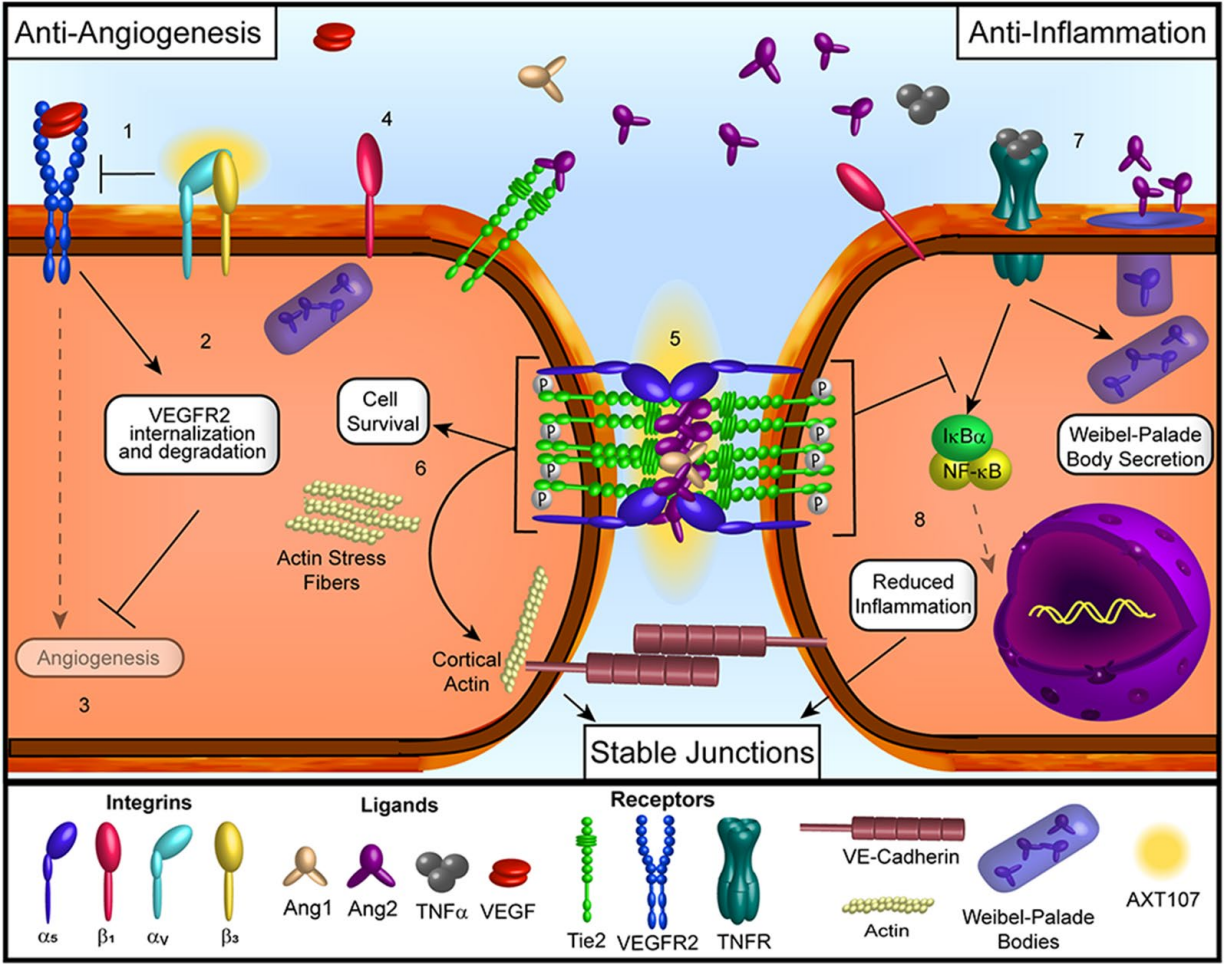
Effects of AXT107-treatment in retinal vascular disease. (1) AXT107 binds to α_v_β_3_ integrins and dissociates them from VEGFR2. (2) The disruption of these interactions directly inhibits the autophosphorylation of VEGFR2 in the presence of VEGF and further reduces VEGFR2 responses by increasing the internalization and degradation of the receptor, overall reducing the angiogenesis responses and induction of vessel permeability (3). (4) AXT107 also binds to α_5_β_1_ integrin heterodimers, which associate with Tie2, and dissociates the subunits. (5) α_5_ integrin and Tie2 then relocate to the endothelial cell–cell junctions and form clusters that can be activated following the binding of either Ang1 or Ang2. (6) Active Tie2 clusters stimulate downstream pathways associated with improved endothelial cell survival and the reorganization of intracellular actin from stress fibers into cortical actin that is distributed around the edges of the cell to stabilize the vasculature. (7) Initial activation of TNFR by TNFα does not seem to be affected by AXT107 treatment, resulting in the release of Ang2 from Weibel-Palade bodies. The released Ang2 contributes to the activation of Tie2 clusters formed following AXT107 treatment. (8) Signaling by the phosphorylated Tie2 clusters prevent the degradation of IκBα molecules, retaining NF-κB within the cytoplasm and blocking the induction of inflammation and the associated effects on vessel permeability, thereby contributing to increased vessel stability

AXT107 suppresses TNFα-induced vascular inflammation in endothelial cells by converting the pro-inflammatory Ang2 into a strong agonist of Tie2 signaling by promoting junctional clustering of Tie2 as explained above, disrupting the synergism between TNFα and Ang2 while also preventing IκBα degradation directly through Tie2 signaling (Fig. 2). The recovery of IκBα prevents NF-κB nuclear localization, thereby blocking NF-κB induced inflammatory responses [55, 93].

Integrin α_v_β_3_ also complexes with VEGF receptor 2 (VEGFR2) to enhance its downstream signaling when ligand-bound by VEGF [101]. In the presence of AXT107, integrin α_v_β_3_ is disrupted and not available to complex with VEGFR2, so that VEGF-bound VEGFR2 phosphorylation is reduced, blocking downstream signaling [92]. In addition, VEGFR2 surface levels are reduced by degradation also contributing to reduced levels of signaling. Consequently, choroidal neovascularization and vascular leakage are inhibited.

AXT107 solution is delivered by intravitreal (IVT) injection in preclinical rabbit and minipig models. In the vitreous AXT107 self-assembles into a small gel-like depot post-IVT injection and settles into the inferior vitreous, away from the visual axis, and dissipates over months [92]. In preclinical studies, AXT107 has been shown to suppress and regress CNV in a laser-induced mouse model [92], suppress subretinal neovascularization in *rho/VEGF* transgenic mice [92], suppress ischemia-induced retinal neovascularization in a mouse model of oxygen-induced ischemic retinopathy [92], suppress VEGF-induced vascular leakage in *rho/VEGF* transgenic mice and in Dutch Belted rabbits [92], and reduce inflammation in mouse model of TNFα-induced inflammation and leukostasis [93]. In Dutch Belted rabbits, anti-permeability activity was still present after 60 days when re-challenged with VEGF in AXT107-treated eyes but not in aflibercept-treated eyes [92]. In an exploratory study with a single IVT injection of AXT107 in a modified Dutch Belted rabbit model of VEGF-induced vascular leakage, the AXT107 demonstrated inhibition of vascular leakage compared to VEGF controls throughout the 12-month duration. In addition, an ongoing PK study in Dutch Belted rabbit demonstrated drug level in the retina for at least 12 months (data on file). Such observations in rabbit eyes—which more closely approximate the size of human eyes than do mouse eyes—suggest the possibility of infrequent dosing of AXT107 in human eyes, which could significantly reduce the treatment burden posed by every 1–3 month retreatment of currently approved anti-VEGF agents for retinal vascular disease.

Clinical development of AXT107 will be initiated in late 2020 with the Investigational New Drug filing of phase 1/2a clinical studies to evaluate the safety and bioactivity of AXT107 in the management of DME, nAMD, and RVO.

Several other compounds that target the Tie2 signaling pathway are in various stages of clinical development (Table 2).

#### Faricimab

Faricimab is a bispecific antibody that binds and blocks both VEGF and Ang2 without any binding to Ang1 [58, 102, 103]. The phase 2 AVENUE trial in eyes with treatment-naïve nAMD showed that faricimab given by IVT injection every 4 weeks (q4wk) provided better best-corrected visual acuity (BCVA) gains than ranibizumab q4wk or the combination of faricimab and ranibizumab each given q4wk at 36 weeks [104]. The phase 2 STAIRWAY trial in eyes with treatment-naïve nAMD showed that faricimab every 12 weeks (q12wk) and every 16 weeks (q16wk) provided comparable BCVA and anatomic outcomes [macular thickness on optical coherence tomography (OCT) imaging] after 1 year of therapy [105]. The phase 2 BOULEVARD trial in DME compared two doses of faricimab to monthly ranibizumab and found that the higher dose faricimab produced BCVA gains that were superior to ranibizumab at 24 weeks with no safety issues identified [106]. Phase 3 trials are underway for nAMD [107, 108] and DME [109, 110].

#### Razuprotafib (formerly AKB-9778)

Razuprotafib is a small molecule inhibitor of VE-PTP. Its inhibition leads to activation of Tie2 pathways and promotes all downstream signaling (thus stabilizing ocular vasculature) by promoting Tie2 phosphorylation, enhancing Ang2-induced Tie2 phosphorylation, and increasing Tie2 phosphorylation in the presence of Ang2 [111]. The drug is dosed by subcutaneous injection twice daily. A phase 1 trial in DME revealed that doses of 15 mg or more resulted in improved BCVA and reductions in OCT central subfield thickness (CST) in some eyes with no systemic safety issues identified [112]. A phase 2 trial in DME compared razuprotafib in combination with ranibizumab to each component as monotherapy [113]. Improvements in BCVA were comparable with ranibizumab and the combination but minimal in the razuprotafib monotherapy group; greater CST reduction was seen in the combo group than either monotherapy group. These results suggested that Tie2 activation with VEGF inhibition reduces DME more than VEGF inhibition alone. No safety issues were identified. Two phase 2 trials were conducted in eyes with nonproliferative DR. In the first (TIME-2a), comparable proportions of eyes manifested a ≥ 2-step improvement in DR scores in the ranibizumab, razuprotafib, and combination groups (8.8%, 10.0%, and 11.4%, respectively) [114]. In the second (TIME-2b), there was no significant difference between razuprotafib and placebo in the proportions of eyes attaining a ≥ 2-step improvement in DR scores [115]. This finding suggested that Tie2 activation requires concomitant VEGF suppression for optimal efficacy. A phase 2 study in eyes with macular edema secondary to RVO was completed but not reported [116]. ARP-1536 is a humanized monoclonal antibody targeting VE-PTP with comparable efficacy to AKB-9778 but with intravitreal administration that is in development [117].

#### Nesvacumab

Nevascumab is a fully human IgG monoclonal anti-Ang2 antibody that binds Ang2 and blocks its binding to Tie2. It was co-formulated with aflibercept for intravitreal injection, and was well tolerated in a phase 1 trial in nAMD and DME with promising effects on BCVA [118] but failed to demonstrate superiority with respect to visual acuity gains in phase 2 studies in nAMD (ONYX) [119] and DME (RUBY) [120] and development was abandoned without phase 3 evaluation [121]. Despite the failure to demonstrate superiority in visual acuity, there were signals of anatomic and durability benefits.

## Summary and Conclusion

Retinal vascular diseases such as nAMD, DME, and macular edema associated with RVO are characterized by the presence of neovascularization and/or increased vascular permeability, and the contribution from inflammatory processes. The VEGF signaling pathway generally regulates angiogenesis while the Tie2 signaling pathway oversees the maturation and stability of the vasculature. Tie2 is a receptor tyrosine kinase with two key peptide ligands: angiopoietin-1 (Ang1) is a receptor *agonist* while angiopoietin-2 (Ang2) is primarily a receptor *antagonist* but can function as an agonist in some circumstances. Activation of the Tie2 pathway leads to downstream signaling that promotes vascular health and stability and decreases vascular permeability and inflammation.

The Tie2 pathway is active in ocular vascular beds, is integral to neovascularization and permeabiity in retinal vascular diseases, and represents a novel therapeutic target for these conditions. AXT107 is a collagen IV—derived synthetic peptide with a dual mechanism of action that involves suppression of VEGF signaling and activation of the Tie2 pathway; these actions are accomplished by AXT107 binding to and disrupting integrin α_v_β_3_ and integrin α_5_β_1_ leading to blockade of the VEGF receptor and rearrangement of cellular Tie2 rendering it susceptible to Ang2 agonism. In animal models, AXT107 suppresses neovascularization, decreases vascular permeability, and reduces inflammation. Other molecules in development to target the Tie2 pathway include faricimab, a bispecific antibody that blocks both VEGF and Ang2 (now in phase 3 trials for nAMD and DME) and razuprotafib, a small molecule inhibitor of the Tie2 inhibitor VE-PTP (effectively enhancing Tie2 activity).

Tie2 activation only modestly impacts angiogenesis on its own but significantly potentiates VEGF suppression; co-regulation of the VEGF and Tie2 signaling pathways has the potential to improve functional and structural outcomes in eyes with retinal vascular diseases.

## Data Availability

Not applicable
